# High-Density Mapping and Candidate Gene Analysis of *Pl*_18_ and *Pl*_20_ in Sunflower by Whole-Genome Resequencing

**DOI:** 10.3390/ijms21249571

**Published:** 2020-12-16

**Authors:** Guojia Ma, Qijian Song, Xuehui Li, Lili Qi

**Affiliations:** 1 Department of Plant Sciences, North Dakota State University, Fargo, ND 58108, USA; guojia.ma@ndsu.edu (G.M.); Xuehui.li@ndsu.edu (X.L.); 2 USDA-Agricultural Research Service, Soybean Genomics and Improvement Laboratory, Beltsville, MD 20705-2350, USA; qijian.song@usda.gov; 3 USDA-Agricultural Research Service, Edward T. Schafer Agricultural Research Center, Fargo, ND 58102-2765, USA

**Keywords:** sunflower, downy mildew, disease resistance, whole-genome resequencing, fine mapping, SNP markers

## Abstract

Downy mildew (DM) is one of the severe biotic threats to sunflower production worldwide. The inciting pathogen, *Plasmopara halstedii,* could overwinter in the field for years, creating a persistent threat to sunflower. The dominant genes *Pl*_18_ and *Pl*_20_ conferring resistance to known DM races have been previously mapped to 1.5 and 1.8 cM intervals on sunflower chromosomes 2 and 8, respectively. Utilizing a whole-genome resequencing strategy combined with reference sequence-based chromosome walking and high-density mapping in the present study, *Pl*_18_ was placed in a 0.7 cM interval on chromosome 2. A candidate gene HanXRQChr02g0048181 for *Pl*_18_ was identified from the XRQ reference genome and predicted to encode a protein with typical NLR domains for disease resistance. The *Pl*_20_ gene was placed in a 0.2 cM interval on chromosome 8. The putative gene with the NLR domain for *Pl*_20_, HanXRQChr08g0210051, was identified within the *Pl*_20_ interval. SNP markers closely linked to *Pl*_18_ and *Pl*_20_ were evaluated with 96 diverse sunflower lines, and a total of 13 diagnostic markers for *Pl*_18_ and four for *Pl*_20_ were identified. These markers will facilitate to transfer these new genes to elite sunflower lines and to pyramid these genes with broad-spectrum DM resistance in sunflower breeding.

## 1. Introduction

Downy mildew (DM) is a devastating sunflower disease throughout the world, particularly in Europe and North America [1,2]. It is incited by the oomycete pathogen *Plasmopara halstedii* (Farl.) Berlese & de Toni, which could overwinter and persist in the soil for 5–10 years. Cool and moist soil favors downy mildew epidemics in sunflower fields. Although sunflower is the field crop that was infected by this DM fungus, other susceptible plants of weeds in the Compositae family, such as marsh elder, could function as reservoirs for this soil-borne fungus (https://www.ag.ndsu.edu/extensionentomology/recent-publications-main/publications/A-1331-sunflower-production-field-guide). DM infection is found mostly in the Northern Great Plains within the U.S., and the disease infected approximately 16% of sunflower fields in 2015 [3]. Substantial yield loss is expected upon DM infection, as severely infected plants will not proceed to growth at the seedling stage with few exceptions in which infected plants could still grow to maturity but not produce viable seeds.

Seed treatment would help DM management to some extent; however, it increases costs with potential harm to the environment. The utilization of resistant hybrids is the first choice to mitigate the negative effect of DM disease. The DM resistance present in sunflower is predominantly identified as a single dominant gene, designated *Pl*, and a total of 36 *Pl* genes, *Pl*_1_*–Pl*_35_, and *Pl_Arg_*, have been reported in sunflower and its wild relatives so far (Supplementary Table S1). Because of the emergence of new *P. halstedii* pathotypes when DM resistance genes (*R* genes) are widely used in sunflower production, most DM *R* genes are no longer effectively resistant to *P. halstedii* infection [4]. Discovering and integrating new resistance genes is essential to ensure commercial hybrids to remain resistant to ever-changing pathogens. 

Sunflower wild species represent a large gene pool for agronomically important traits including male sterile cytoplasm/male fertility restorers, disease resistance, abiotic tolerance, and herbicide resistance, which are widely explored in sunflower breeding [5]. Most DM *R* genes can be traced to their wild origins, mainly from wild *Helianthus annuus* and *Helianthus argophyllus* (Supplementary Table S1). The two DM *R* genes, *Pl*_18_ and *Pl*_20_, were identified from *H. argophyllus* accessions of PI 494573 and PI 494578 originally collected in 1984 from Texas, U.S., respectively, with a broad spectrum of resistance to new races of *P. halstedii* [1,6,7]. Molecular mapping has placed *Pl*_18_ in a 1.5 cM interval flanked by SSR markers, CRT214 and ORS203, on chromosome 2 of the sunflower genome [7]. The breeding project successfully introgressed *Pl*_18_ from sunflower wild species into cultivated sunflower. The oilseed sunflower germplasm HA-DM1 carrying *Pl*_18_ was released to the public and was highly resistant to all *P. halstedii* races identified in the U.S. [4,8].

Biparental mapping efforts placed *Pl*_20_ in a 1.8 cM interval on the upper end of sunflower chromosome 8 within a gene cluster harboring the five DM *R* genes, *Pl*_1_, *Pl*_2_, *Pl*_6_, *Pl*_7_, and *Pl*_15,_ and the two rust *R* genes, *R*_1_ and *R*_15_ [1,9,10,11,12]. HA-DM7 is an oilseed maintainer line introgressed with *Pl*_20_ released in 2019. The flanking markers of *Pl*_18_ and *Pl*_20_ were physically positioned *Pl*_18_ and *Pl*_20_ within intervals of 780 kb and 1 Mb on chromosomes 2 and 8 in the XRQ genome, respectively. Traditional biparental mapping can chromosomally locate genes efficiently; however, the relatively large gene interval could still impair its use in breeding programs, as recombination events would be inevitable within the defined intervals. Diagnostic molecular markers resulting from high-resolution mapping would be helpful to integrate new resistance gene through marker-assisted selection (MAS) in an accurate manner.

Currently, two sunflower lines, HA412-HO and XRQ, have been whole-genome sequenced, assembled and annotated and are publicly available, each utilizing different sequencing techniques (https://www.heliagene.org/). The HA412-HO whole-genome sequence was assembled based on Illumina HiSep and Roche 454 second-generation sequencing (SGS) data with short reads, while the XRQ whole-genome sequence was assembled based on PacBio RS II data with an average read length of 10.3 kb (N50 of 13.7 kb) [13]. The two reference genome sequences provide alternative tools to study marker-trait associations, to accelerate the fine-scale genetic mapping of important sunflower genes, and to identify diagnostic markers near or even within genes of interest. With the rapid progress and innovation of sequencing technologies with reduced cost, whole-genome sequencing (WGS) has become the most global approach to enable high-resolution trait mapping, candidate gene discovery, and breeding applications in plants. In this study, we performed WGS of two sunflower lines, HA-DM1 and HA-DM7, which carry the DM *R* genes *Pl*_18_ and *Pl*_20_, respectively. Sequence information was used to develop SNP markers closely linked and diagnostic to *Pl*_18_ and *Pl*_20_ and to identify gene candidates associated with resistance to DM.

## 2. Results

### 2.1. Saturation Mapping of Pl_18_

**Figure 1:**
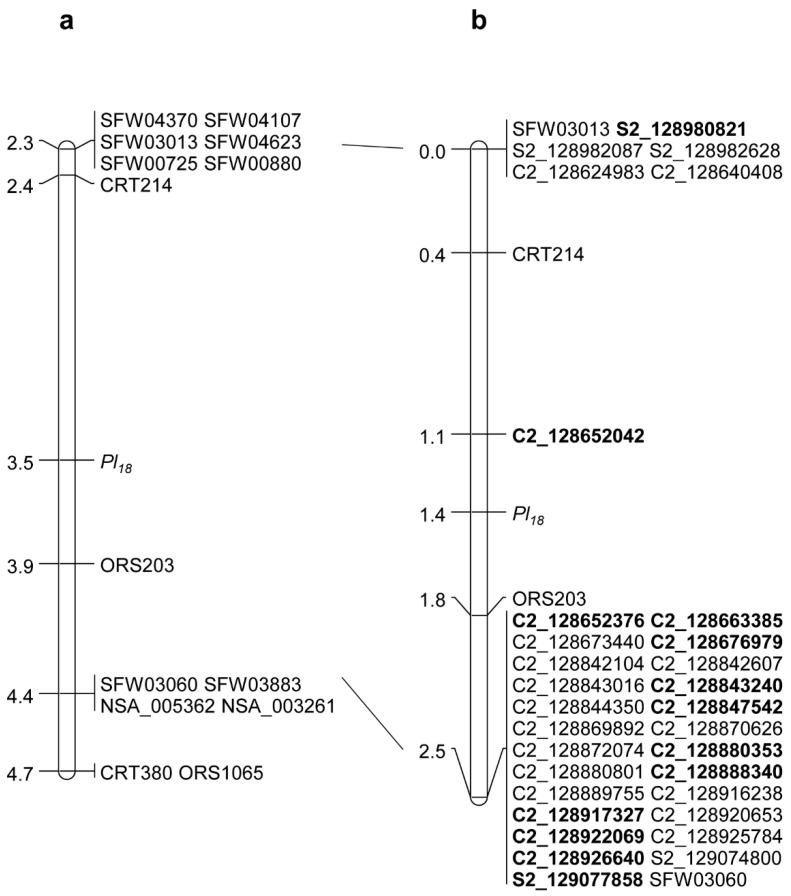
*Pl*_18_ genetic maps. (**a**) *Pl*_18_ basic map [7]; (**b**) *Pl*_18_ saturation map. The diagnostic markers for *Pl*_18_ are shown in bold.

The DM *R* gene *Pl*_18_ was previously placed in an interval of 1.5 cM genetic distance flanked by the SSR markers CRT214 and ORS203 on chromosome 2 [7]. Neither SSR marker can be aligned to the reference genomes, and their physical positions on the genome are unknown. Alternatively, two SNP markers, SFW03013 closely linked to CRT214 at 0.1 cM and SFW03060 closely linked to ORS203 at 0.5 cM, were selected, delimiting *Pl*_18_ to a physical interval of 780,432 bp between 128,511,770–129,292,202 bp in the XRQ genome and 100,508 bp between 128,982,063–129,082,571 bp in the HA412-HO genome (Table 1, Figure 1a). A total of 150 SNPs were selected based on SNPs/InDels between HA-DM1 carrying *Pl*_18_ and the two reference genomes in the targeted region of chromosome 2. Forty-nine were selected from the HA412-HO genome, and 101 were selected from the XRQ genome. Forty-three SNP markers showed polymorphisms between HA 89 and HA-DM1 and were used to genotype the initial 142 BC_1_F_2_ individuals. Thirty-one SNP markers were mapped around *Pl*_18_, and SNP C2_128652042 was the only marker mapped to the *Pl*_18_ interval between SSRs CRT214 and ORS203, which is 0.3 cM distal to *Pl*_18_ (Figure 1b). A large marker cluster with 26 co-segregating SNPs was proximal to ORS203 at a 0.7 cM genetic distance.

Among 31 SNP markers mapped to the *Pl*_18_ interval between SNP markers SFW03013 and SFW03060, only four, S2_128980821, S2_128982087, S2_129074800, and S2_129077858, were from the HA412-HO genome, and their physical positions on both HA412-HO and XRQ assemblies were in agreement with their genetic positions (Table 1, Figure 1b). However, no SNPs designed from the 93 kb region between S2_128982087 and S2_129074800 were mapped. The remaining 27 mapped SNPs were all from the XRQ genome (Figure 1b). All of the markers were physically in accordance with their genetic positions on the XRQ assembly; however, these SNPs did not align to the target region between 128,982,147 and 129,082,571 bp delimited by SFW03013 and SFW03060 in the HA412-HO assembly (Table 1). Among 27 mapped SNPs derived from a 302 kb region between C2_128624983 and C2_128926640 of XRQ, three aligned to 132.74–132.75 kb positions of HA412-HO, two to 97.02–97.03 kb positions, 21 to 129.43–129.67 kb positions, and one did not align to chromosome 2 (Table 1). The results suggest that the 93 kb region between S2_128982087 and S2_129074800 in HA412-HO may not be assembled correctly, which may also explain why no SNPs designed from this region were mapped.

### 2.2. Saturation and Fine Mapping of Pl_20_

**Figure 2:**
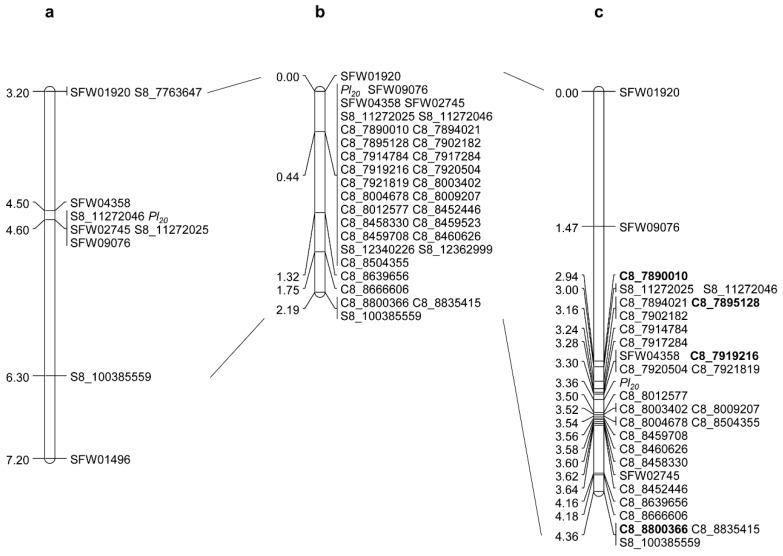
*Pl*_20_ genetic maps. (**a**) *Pl*_20_ basic map [1]; (**b**) *Pl*_20_ saturation map; (**c**) *Pl*_20_ fine map. The diagnostic markers for *Pl*_20_ are shown in bold.

*Pl*_20_ was previously placed within the 2.5 Mb interval of 11,271,845–13,781,094 bp on chromosome 8 of the HA412-HO reference genome flanked by SNP markers S8_11272046 and SFW01496 [1]. A total of 244 SNP markers were selected from the SNP/InDel calling of whole-genome sequencing of HA-DM7 with HA412-HO and XRQ on chromosome 8; 84 from the HA412-HO genome covering a region of 447 kb (12,254,876–12,701,559 bp), and 160 from the XRQ genome covering a region of approximately 1.0 Mb (7,890,010–8,906,527 bp). The 244 SNP markers potentially surrounding *Pl*_20_ were tested between parents HA 89 and HA-DM7, and 25 showed polymorphism, including 2 from HA412-HO and 23 from XRQ. These 25 polymorphic markers were further genotyped with 114 BC_1_F_2_ individuals of the original population, and all were mapped around *Pl*_20_ (Figure 2b). A marker cluster with 26 SNPs co-segregated with *Pl*_20_, and SNP marker C8_8639656 was proximal to *Pl*_20_ at a 0.88 cM genetic distance.

To dissect the above marker cluster and to develop a high-density map of *Pl*_20_, a large population with the 2,485 BC_1_F_3_ individuals selected from the BC_1_F_3_ families heterozygous for *Pl*_20_ was genotyped using two flanking SNP markers, SFW01920 and S8_100385559. A total of 214 BC_1_F_3_ recombinants were identified in the target region delimited by the two markers and advanced to the next generation for DM testing of the recombinant families.

Twenty-two co-dominant SNP markers in the saturation map were selected to genotype the 214 recombinants identified from the above large population. Additionally, seven previously mapped SNP markers, SFW01920, SFW09076, S8_11272025, S8_11272046, SFW04358, SFW02745, and S8_100385559, within the *Pl*_20_ region were also included in the fine mapping [1]. The combined phenotype and marker data of the recombinants positioned *Pl*_20_ to a 0.20 cM interval, flanked by SNP markers C8_7921819 (0.06 cM) and C8_8012577 (0.14 cM) (Figure 2c). This genetic region corresponds to a 91.2 kb segment in the XRQ assembly (Table 2).

### 2.3. Identification of Candidate Genes for Pl_18_ and Pl_20_

In the high-density map, all of the newly developed SNP markers were mapped around *Pl*_18_ and physically located in a 780 kb region between 128,511,770 and 129,292,202 bp on chromosome 2 of the XRQ assembly (Table 1). These SNP markers are genetically and physically consistent with the position in the XRQ genome, and thus the 780 kb genomic sequences in the target region were analyzed from the XRQ database (https://www.heliagene.org/HanXRQ-SUNRISE/). Seven highly confident genes were found in the target region, and one putative gene, HanXRQChr02g0048181, was predicted to code a powdery mildew resistance protein with the typical disease resistance gene domain of nucleotide binding and leucine-rich repeat (NLR) (Table 3). This gene is located from 128,920,787 to 128,926,787 bp along chromosome 2 with a length of 6 kb. Its genetic and physical positions, as well as its functional domains and predicted functions, support it as a candidate gene for *Pl*_18_. 

Similarly, an approximately 610 kb genomic sequence (7,894,928–8,504,555 bp) between SNP markers C8_7895128 and C8_8504355 of chromosome 8 was extracted from the XRQ database for annotation, and seven genes in this region were identified. Out of seven genes, HanXRQChr08g0210051 has a typical NLR domain, and HanXRQChr08g0210151 has a leucine-rich repeat domain (Table 3). HanXRQChr08g0210051 was located from 8,010,685 to 8,035,718 bp on chromosome 8 with a length of 25 kb, which falls to the *Pl*_20_ gene interval between SNP markers C8_7921819 and C8_8012577 and could be a candidate gene for *Pl*_20_ (Table 3,). HanXRQChr08g0210151 was located from 8,408,566 to 8,411,305 bp on chromosome 8 with a length of 2.7 kb (Table 3).

### 2.4. Development of Diagnostic Markers for Pl_18_ and Pl_20_

**Figure 3:**
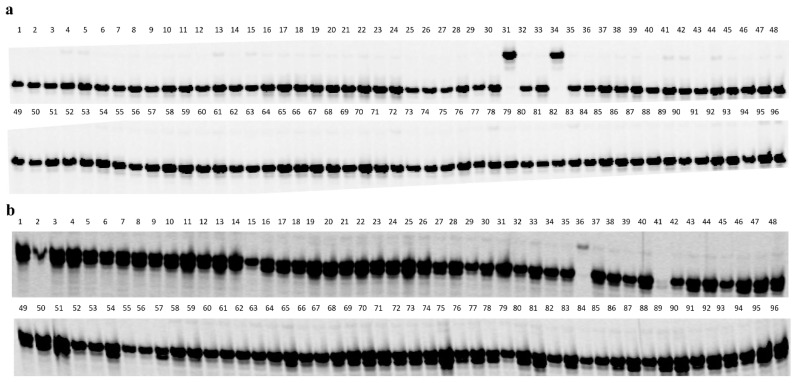
The polymerase chain reaction (PCR) amplification pattern of 96 selected sunflower lines with *Pl*_18_ and *Pl*_20_ diagnostic single nucleotide polymorphism (SNP) markers. The names and pedigrees of 96 selected sunflower lines (lanes) are listed in Supplementary Table S2. (**a**) PCR amplification pattern with SNP marker C2_128922069 diagnostic for *Pl*_18_. Lane 31: HA-DM1, and lane 34: HA-DM4, both have the *Pl*_18_ gene and show the *Pl*_18_ marker allele. (**b**) PCR amplification pattern with SNP marker C8_7895128 diagnostic for *Pl*_20_. Lane 36: HA-DM7 with the *Pl*_20_ gene.

The 31 new SNP markers mapped to *Pl*_18_ in the saturation map were first tested in six sunflower lines, including four resistant lines, HA 458 (*Pl*_17_), HA-DM1 (*Pl*_18_), RHA 340 (*Pl*_8_), and RHA 464 (*Pl_Arg_*), and two susceptible lines, HA 89 and CONFSCLB1. Twenty-two of them showed a unique PCR pattern in HA-DM1 in contrast to the other three resistant and two susceptible lines and were further genotyped in an evaluation panel with 96 selected sunflower lines to determine their specificity in the sunflower population and to assess their potential in MAS for *Pl*_18_ (Supplementary Table S2). Thirteen of the 22 SNP markers could differentiate *Pl*_18_ from other reported *Pl* genes, including *Pl_Arg_*, *Pl*_1_*–Pl*_3_, *Pl*_6_*–Pl*_13_, *Pl*_15_*–Pl*_21_, *Pl*_33_, and *Pl*_34,_ in the selected sunflower lines (Table 1; Figure 3a). *Pl*_18_-introgressed lines, HA-DM1 and HA-DM4, show unique *Pl*_18_ marker alleles, differentiating them from other sunflower lines (Figure 3a).

Eight SNP markers fine mapped around *Pl*_20_ were selected to test for specificity in the evaluation panel of 96 selected sunflower lines, and four showed unique patterns in HA-DM7 (*Pl*_20_-introgressed line), in contrast to others without the *Pl*_20_ gene (Table 2; Figure 3b). These *Pl*_18_ and *Pl*_20_ diagnostic markers identified are of great importance and usefulness to assist selection for both genes in sunflower breeding programs.

## 3. Discussion

Similar to other disease resistance genes in crops, DM *R* genes were found mostly in clusters along sunflower chromosomes 1, 4, 8, and 13. Out of 29 mapped DM *R* genes, eight in chromosome 1 included sub-cluster I of *Pl_Arg_*, *Pl*_23_, *Pl*_24_ and *Pl*_35_, and sub-cluster II of *Pl*_13_, *Pl*_14_, *Pl*_16_ and *Pl*_25_, six each in chromosome 4 (*Pl*_17_, *Pl*_19_, *Pl*_27_–*Pl*_29_, and *Pl*_33_) and 8 (*Pl*_1_, *Pl*_2_, *Pl*_6_, *Pl*_7_, *Pl*_15_, and *Pl*_20_), and seven (*Pl*_5_, *Pl*_8_, *Pl*_21_, *Pl*_22_, *Pl*_31_, *Pl*_32_, and *Pl*_34_) in chromosome 13 (Supplementary Table S1). Most of the clusters are characterized as having NLR motifs, which are the major class of disease-resistant genes found in flowering plants [14,15,16,17]. In the present study, the predicted candidate genes for both *Pl*_18_ and *Pl*_20_ are NLR-type genes, which are strongly indicative of disease-resistant genes, as found in chickpea [18], soybean [19], maize [20], wheat [21], and rice [22].

Genomic regions encompassing NLR clusters are very likely attributed to duplications in which chromosome doubling is presumed to occur during sunflower evolution [13]. Three models have been proposed for duplicated genes, i.e., pseudogenized (loss of regulatory sub-function), sub-functionalized (partitioning of the function between daughter copies) and/or neo-functionalized (functional diversification) [23]. With a fair chance that some of the NLR-involved genes within the clusters were pseudogenized after duplication and during the interaction with pathogens, it is possible that the genes conferring resistance might not be present in the reference genome of XRQ, even though the typical NLR motifs were present. To validate their candidacy, the candidate genes predicted from the reference genome need to be landed to the resistance donor lines, followed by functional characterization. Because of the short reads from the Illumina whole-genome sequencing and high level of repetitive sequences in the sunflower genome, it is difficult to assemble a scaffold covering the entire gene sequence from the sequenced donor line because most of the contigs (81%) and scaffolds (86%) assembled in a previous study ranged between 100 and 500 bp, and only 6% of contigs and 8% of scaffolds were over 1 kb, leaving a large number of gaps [24]. The physical localization of each *Pl*_18_ and *Pl*_20_ to a region less than 100 kb on chromosomes 2 and 8, respectively, in the present study represents a significant step toward the final cloning and functional characteristics of these *R* loci. PacBio long-read target region sequencing provides a powerful tool to capture these two genomic regions harboring the candidate genes. This technology combined with analysis of ethyl methanesulfonate (EMS)-induced mutants will allow us to distinguish among the possibilities and to uncover the genetic and molecular basis of DM disease resistance in sunflower.

Unlike most DM *R* genes located on clusters in sunflower chromosomes 1, 4, 8, and 13 mentioned above, only two genes, *Pl*_18_ and *Pl*_26_, were mapped to sunflower chromosome 2 [25]. As a result of the limited mapping resolution and lack of recombination in the region, *Pl*_26_ was placed in a relatively larger interval of 114 Mb physically on XRQ (26,000,000–140,000,000 bp), while *Pl*_18_ was located within the 128,640,208–129,297,096 bp interval of chromosome 2. *Pl*_18_ originated from *H. argophyllus* accession PI 494573 collected from Texas, U.S., while *Pl*_26_ originated from *H. annuus* HAS103. Although *Pl*_18_ falls within the large region encompassing *Pl*_26_ on chromosome 2, their different origins suggest that they are different resistance genes. Further fine mapping of *Pl*_26_ would elucidate the genetic relationship of the two genes.

Chromosome 8 of sunflower represents the largest and most important NLR cluster, including 54 NLR loci [26]. The DM *R* gene cluster was located in the first and largest sub-cluster containing *Pl*_1_, *Pl*_2_, *Pl*_6_, *Pl*_7_, *Pl*_15_ and *Pl*_20_ and two rust *R* genes *R*_1_ and *R*_15_ [1]. *Pl*_20_ originated from *H. argophyllus* is different from other *Pl* genes in the cluster in which *Pl*_1_, *Pl*_2_ and *Pl*_6_ were from wild *H. annuus*, *Pl*_7_ was from *H. praecox*, and *Pl*_15_ was identified from an Argentinian restorer inbred line [1,27,28,29,30,31]. *Pl*_20_ was immune to all *P. halstedii* races identified in North America, including those predominant and virulent races; however, the remaining *Pl* genes, except *Pl*_15_ in the cluster, have already been overcome by some or all of the identified *P. halstedii* races. In the current study, four SNP markers, C8_7890010, C8_7895128, C8_7919216, and C8_8800366, showed unique PCR patterns in HA-DM7 (*Pl*_20_-introgressed line), distinguishing it from other *Pl* genes in the cluster. All the findings suggest that *Pl*_20_ is a novel and most effective DM *R* gene that serves as a powerful resistance resource for durable DM control in sunflower.

MAS has been extensively used in modern plant breeding, especially for traits controlled by single genes. The success of MAS is influenced by the relationship between the markers and the genes of interest, and it is important that the recombination frequency between the target gene and the marker is as low as possible. Our high-resolution genetic maps and the diagnostic markers for *Pl*_18_ and *Pl*_20_ developed will be useful tools facilitating the transfer of these new genes to elite sunflower lines in breeding programs.

## 4. Materials and Methods

### 4.1. Mapping Populations and Evaluation Panel

The *Pl*_18_ F_2_ initial mapping population was created from a cross of nuclear male sterile (NMS) HA 89 × *H. argophyllus* accession PI 494573, and F_1_ was backcrossed with normal HA 89, including 142 BC_1_F_2_ individuals [7]. Similarly, the *Pl*_20_ F_2_ initial mapping population was developed from a cross of NMS HA 89 × *H. argophyllus* accession PI 494578, including 114 BC_1_F_2_ individuals [7]. The *H. argophyllus* accessions, PI 494573 and PI 494578, were found to be resistant to new races of *P. halstedii*, while HA 89 is susceptible to all *P. halstedii* races [6]. The germplasms HA-DM1 with *Pl_1_*_8_ and HA-DM7 with *Pl*_20_ were developed and released in 2015 and 2019, respectively [8], and were each used as the *Pl*_18_ and *Pl*_20_ donor lines for whole-genome resequencing for high-density mapping of both *R* genes.

For fine mapping of *Pl*_20_, recombinants were screened from 2,485 BC_1_F_3_ individuals selected from the previously characterized BC_1_F_2:3_ families heterozygous for *Pl*_20_. Each selected heterozygous F_3_ family equates to a segregating F_2_ population for the *Pl*_20_ gene.

The specificity of diagnostic *Pl*_18_ and *Pl*_20_ SNP markers was tested in the sunflower evaluation panel, consisting of 96 sunflower inbred lines of diverse origins. This panel includes 24 and 17 lines with different DM and rust *R* genes, respectively (Supplementary Table S2).

### 4.2. SNP Marker Development from Whole-Genome Resequencing

HA-DM1 (*Pl*_18_) and HA-DM7 (*Pl*_20_) were sequenced at the whole-genome level separately by CD Genomics Inc. using the Illumina HiSeq sequencing platform. According to the protocols, genomic DNA of HA-DM1 and HA-DM7 was first checked for quality to ensure that the level of contamination and degradation was low enough to meet their requirements. The quality genomic DNA was sheared with the use of an S/E2*10* focused ultrasonicator (Covaris, Woburn, MA, USA) for library construction. Qualified libraries for either *Pl*_18_ or *Pl*_20_ were pooled and subjected to sequencing at 40 × genome coverage. The raw reads containing adaptors, reads with >1% ambiguous bases, and reads with low quality (greater than 50% bases less than 15 Q score) were removed and excluded for further analysis. The clean reads were aligned to the two reference genomes of XRQ (https://www.heliagene.org/HanXRQ-SUNRISE/) and HA412-HO (https://www.heliagene.org/HA412.v1.1.bronze.20141015/), respectively. After filtering of low-quality reads, a total of 1,166,680,112 (99.09%) HA-DM1 reads and 1,023,555,572 (98.74%) HA-DM7 reads were mapped to the references XRQ and HA412-HO, respectively. All SNPs and InDels were identified using the mapped reads. The SNP markers were named with prefix C2, S2, C8 or S8 followed by a number representing the physical position of the SNPs along either chromosome 2 or 8 of each reference genome assembly. C2 and C8 represent the SNPs from chromosomes 2 and 8 of the XRQ reference genome, while prefixes S2 and S8 represent the SNPs from chromosomes 2 and 8 of the HA412-HO reference genome.

### 4.3. Genotyping of PCR-Based SNP Markers and Linkage Analysis

Polymerase chain reaction (PCR)-based SNP primers were designed with the Primer 3 program, and specific mismatches and length polymorphisms for SNP primers were created (Supplementary Table S3) as described by Qi et al. [32] and Long et al. [33] based on SNP flanking sequences (Supplementary Tables S4 and S5). PCR for SNPs was conducted as described by Ma et al. [34], and amplicons were separately scored on a 6.5% polyacrylamide gel using an IR2 4300/4200 DNA analyzer (LI-COR, Lincoln, NE, USA).

The chi-square (χ^2^) test was performed on genotyping data of each marker to test for goodness-of-fit to the Mendelian segregation ratio, i.e., 1:3 for dominant markers and 1:2:1 for co-dominant markers. Upon the exclusion of those unfitted, markers fitting Mendelian ratios were linkage analyzed with either *Pl*_18_ or *Pl*_20_ phenotyping data using JoinMap 4.1 software in which a regression mapping algorithm and Kosambi’s mapping function were selected [35]. The cutoffs of linkage analysis among markers were set at a likelihood of odds (LOD) ≥ 3.0 and maximum genetic distance ≤ 50 centimorgans (cM).

### 4.4. Phenotypic Evaluation of Recombinants

*Pl*_20_ recombinants identified with the respective flanking markers were tested for DM resistance using the *P. halstedii* isolate of race 734, together with their introgressed line HA-DM7 and susceptible parent HA 89, using the whole seedling immersion method as described by Gulya et al. [36] and Qi et al. [32]. Briefly, approximately 40 seeds from each recombinant family were germinated and inoculated with the *P. halstedii* isolate of race 734 after 2–3 days, and at least 30 seedlings for each recombinant family were evaluated. Susceptible seedlings showed sporulation on cotyledons and true leaves, and resistant seedlings showed no sporulation. The genotype of each recombinant was determined as homozygous susceptible if all seedlings in the recombinant family showed sporulation on cotyledons and true leaves, homozygous resistant if none of the seedlings exhibited sporulation, and segregating if some seedlings showed sporulation on cotyledons and true leaves while some showed no sporulation.

## Figures and Tables

**Table 1:** Genetic and physical positions of markers linked to *Pl*_18_ on the saturation map of sunflower chromosome 2.

Marker	No. Recombination	Genetic Distance (cM)	Physical Position on XRQ Assembly		Physical Position on HA412-HO Assembly	
			Start	End	Start	End
**S2_128980821**		0.0	128,510,328	128,510,728	128,980,621	128,981,021
S2_128982087	0	0.0	128,511,594	128,511,994	128,981,887	128,982,287
SFW03013	0	0.0	128,511,854	128,511,770	128,982,147	128,982,063
S2_128982628	0	0.0	128,512,135	128,512,535	128,982,428	128,982,828
C2_128624983	0	0.0	128,624,783	128,625,183	132,735,799	132,736,199
C2_128640408	0	0.0	128,640,208	128,640,608	132,741,177	132,741,577
CRT214	1	0.4	NA	NA	NA	NA
**C2_128652042**	2	0.7	128,651,842	128,652,242	NA	NA
*Pl*_18_	1	0.3	-	-	-	-
ORS203	1	0.4	NA	NA	NA	NA
**C2_128652376**	2	0.7	128,652,176	128,652,576	132,753,709	132,753,885
**C2_128663385**	0	0.7	128,663,185	128,663,585	97,034,302	97,033,902
C2_128673440	0	0.7	128,673,240	128,673,640	97,024,972	97,024,572
**C2_128676979**	0	0.7	128,676,779	128,677,179	97,021,432	97,021,031
C2_128842104	0	0.7	128,841,904	128,842,304	129,433,726	129,434,126
C2_128842607	0	0.7	128,842,407	128,842,807	129,434,224	129,434,624
C2_128843016	0	0.7	128,842,816	128,843,216	129,434,633	129,435,033
**C2_128843240**	0	0.7	128,843,040	128,843,440	129,434,857	129,435,257
C2_128844350	0	0.7	128,844,150	128,844,550	129,435,967	129,436,367
**C2_128847542**	0	0.7	128,847,342	128,847,742	129,646,291	129,646,691
C2_128869892	0	0.7	128,869,692	128,870,092	129,451,579	129,451,979
C2_128870626	0	0.7	128,870,426	128,870,826	129,452,313	129,452,713
C2_128872074	0	0.7	128,871,874	128,872,274	129,670,656	129,670,912
**C2_128880353**	0	0.7	128,880,153	128,880,553	129,545,140	129,545,540
C2_128880801	0	0.7	128,881,001	128,881,401	129,461,631	129,462,020
**C2_128888340**	0	0.7	128,888,140	128,888,540	129,468,821	129,469,221
C2_128889755	0	0.7	128,889,555	128,889,955	129,470,227	129,470,627
C2_128916238	0	0.7	128,916,038	128,916,438	129,497,380	129,497,780
**C2_128917327**	0	0.7	128,917,127	128,917,527	129,498,469	129,498,869
C2_128920653	0	0.7	128,920,453	128,920,853	129,501,790	129,502,189
**C2_128922069**	0	0.7	128,921,869	128,922,269	129,503,206	129,503,605
C2_128925784	0	0.7	128,925,584	128,925,784	129,507,004	129,507,404
**C2_128926640**	0	0.7	128,926,440	128,926,840	129,507,860	129,508,260
S2_129074800	0	0.7	129,300,154	129,299,754	129,074,600	129,074,800
**S2_129077858**	0	0.7	129,297,096	129,296,696	129,077,658	129,078,058
SFW03060	0	0.7	129,292,083	129,292,202	129,082,571	129,082,452

The diagnostic SNP marker for *Pl*_18_ is shown in bold. NA: not available.

**Table 2:** Genetic and physical positions of markers linked to *Pl*_20_ on the fine map of sunflower chromosome 8.

Marker	No. Recombination	Genetic Distance (cM)	Physical Position on XRQ Assembly (bp)	Physical Position on HA412-HO Assembly (bp)
SFW01920 ^†^		0.00	855,100–855,219	8,626,529–8,626,648
SFW09076 ^†^	73	1.47	6,259,434–6,259,553	9,317,681–9,317,800
**C8_7890010**	73	1.47	7,889,810–7,890,210	11,328,554–11,328,954
S8_11272025 ^†^	3	0.06	7,890,621–7,891,021	11,271,825–11,272,225
S8_11272046 ^†^	0	0.00	7,890,600–7,891,000	11,271,846–11,272,246
C8_7894021	8	0.16	7,893,821–7,894,221	11,268,522–11,268,922
**C8_7895128**	0	0.00	7,894,928–7,895,328	11,323,742–11,324,142
C8_7902182	0	0.00	7,901,982–7,902,382	11,262,375–11,262,775
C8_7914784	4	0.08	7,914,584–7,914,984	11,251,806–11,252,206
C8_7917284	2	0.04	7,917,084–7,917,484	11,249,306–11,249,706
SFW04358 ^†^	1	0.02	6,438,630–6,438,749	10,072,538–100,72,657
**C8_7919216**	0	0.00	7,919,016–7,919,416	11,247,370–11,247,773
C8_7920504	0	0.00	7,920,304–7,920,704	11,246,082–11,246,482
C8_7921819	0	0.00	7,921,619–7,922,019	11,244,767–11,245,167
*Pl*_20_	3	0.06	–	–
C8_8012577	7	0.14	8,012,377–8,012,777	9,689,417–9,689,678
C8_8003402	1	0.02	8,003,202–8,003,602	9,680,535–9,680,935
C8_8009207	0	0.00	8,009,007–8,009,407	10,853,046–10,853,441
C8_8004678	1	0.02	8,004,478–8,004,878	9,681,811–9,682,211
C8_8504355	0	0.00	8,504,155–8,504,555	10,905,758–10,906,158
C8_8459708	1	0.02	8,459,508–8,459,908	12,109,436–12,109,838
C8_8460626	1	0.02	8,460,426–8,460,826	12,226,272–12,226,515
C8_8458330	1	0.02	8,458,130–8,458,530	12,108,058–12,108,458
SFW02745 ^†^	1	0.02	8,456,520–8,456,639	11,614,201–11,614,082
C8_8452446	1	0.02	8,452,246–8,452,646	11,620,100–11,620,414
C8_8639656	26	0.52	8,639,456–8,639,856	12,827,866–12,828,204
C8_8666606	1	0.02	8,666,406–8,666,806	12,796,693–12,797,102
**C8_8800366**	9	0.18	8,800,166–8,800,566	13,920,131–13,920,531
C8_8835415	0	0.00	8,835,215–8,835,615	13,960,633–13,961,033
S8_100385559 ^†^	0	0.00	8,907,619–8,908,019	100,385,359–100,385,759

^†^ Mapped previously in Ma et al. 2017 [1]. The diagnostic SNP marker for *Pl*_20_ is shown in bold.

**Table 3:** Predicted genes in the intervals of *Pl*_18_ *and Pl*_20_ from the XRQ annotation.

Genes	Definition	Physical Position	Length (bp)
For *Pl*_18_			
HanXRQChr02g0048101	Probable mog1/PsbP/DUF1795-like photosystem II reaction center PsbP family protein	128,508,926...128,514,561	5636
HanXRQChr02g0048111	Putative glycoside hydrolase family 17; Glycoside hydrolase superfamily	128,551,551...128,553,503	1953
HanXRQChr02g0048131	Putative alcohol dehydrogenase superfamily, zinc-type; L-threonine 3-dehydrogenase; NAD(P)-binding domain	128,639,137...128,639,993	857
HanXRQChr02g0048141	Putative alcohol dehydrogenase superfamily, zinc-type; GroES-like	128,642,764...128,642,967	204
HanXRQChr02g0048151	Putative alcohol dehydrogenase superfamily, zinc-type; GroES-like	128,651,738...128,652,649	912
HanXRQChr02g0048171	Putative NAC domain	128,841,438...128,843,598	2161
HanXRQChr02g0048181	Putative NB-ARC; Powdery mildew resistance protein, RPW8 domain; P-loop containing nucleoside triphosphate hydrolase; Leucine-rich repeat domain, L domain-like	128,920,787...128,926,787	6001
For *Pl*_20_			
HanXRQChr08g0210011	Probable GYF domain-containing protein	7,910,378...7,917,771	7394
HanXRQChr08g0210051	Putative NB-ARC; Toll-like receptor; P-loop containing nucleoside triphosphate hydrolase; Leucine-rich repeat domain, L domain-like	8,010,685...8,035,718	25,034
HanXRQChr08g0210081	Putative tify domain; CO/COL/TOC1, conserved site	8,273,535...8,277,186	3652
HanXRQChr08g0210111	Putative bifunctional inhibitor/plant lipid transfer protein/seed storage helical domain	8,306,497...8,309,225	2729
HanXRQChr08g0210131	Probable CONTAINS InterPro DOMAIN/s: WW/Rsp5/WWP (InterPro:IPR001202)	8,325,909...8,327,735	1827
HanXRQChr08g0210141	Probable B-box type zinc finger protein with CCT domain	8,398,470...8,403,825	5356
HanXRQChr08g0210151	Putative leucine-rich repeat-containing N-terminal, plant-type; Leucine-rich repeat domain, L domain-like	8,408,566...8,411,305	2740

## Supplementary Materials

The following are available online at https://www.mdpi.com/1422-0067/21/24/9571/s1, Table S1. Originating species of designated downy mildew resistance genes; Table S2. Evaluation panel of 96 sunflower lines used to determine the specificity of Pl18 and Pl20 markers; Table S3. Primer sequences of SNP markers mapped in the present study; Table S4. Sequences of SNP markers mapped around Pl18 in the present study (The diagnostic SNP markers for Pl18 are shown in bold); Table S5. Sequences of SNP markers mapped around Pl20 in the present study (The diagnostic SNP markers for *Pl*_20_ are shown in bold). Reference [37,38,39,40,41,42,43,44,45,46,47,48,49,50,51,52,53,54,55,56,57,58,59] are cited in supplementary materials part.
